# Multi-omics landscape of lung mycobiome dysbiosis: *Candida albicans* drives the invasive progression of lung adenocarcinoma

**DOI:** 10.3389/fmicb.2026.1811749

**Published:** 2026-04-15

**Authors:** Haoshuai Yang, Lingyu Yan, Hongxiang Feng, Fei Qi, Fanjia Kong, Qiduo Yu, Weijie Zhu, Chaoyang Liang, Jin Zhang, Zhenrong Zhang

**Affiliations:** Department of Thoracic Surgery, China-Japan Friendship Hospital, Beijing, China

**Keywords:** *Candida albicans*, invasive progression, lung adenocarcinoma, multi-omics, mycobiome, tumor microenvironment

## Abstract

The transition from minimally invasive adenocarcinoma (MIA) to invasive adenocarcinoma (IAC) marks a decisive turning point in lung cancer progression. While the bacterial microbiome is a recognized component of the tumor microenvironment, the specific contribution of the lung mycobiome to this invasive shift remains largely obscure. Using an integrated multi-omics approach, this study maps the fungal ecosystem dynamics across the MIA-to-IAC spectrum. Notably, invasive tissues exhibited a significant elevation in fungal diversity, a finding that stands in sharp contrast to the traditional view of disease-associated microbial loss. We identified *Candida albicans* as the pivotal biomarker distinguishing invasive from indolent lesions. Crucially, functional integration reveals that this fungal enrichment is not merely an association but appears to actively orchestrate a pro-tumorigenic shift in the host’s immune and metabolic landscape. These findings uncover a novel fungal-driven mechanism of tumor invasiveness, suggesting that the lung mycobiome serves as both a hidden driver of progression and a valuable target for early therapeutic intervention.

## 1 Introduction

Lung adenocarcinoma (LUAD) is the primary pathological subtype of non-small cell lung cancer (NSCLC) (Siegel et al., 2026). In clinical practice, its pathological spectrum encompasses a continuous progression from adenocarcinoma *in situ* (AIS) to minimally invasive adenocarcinoma (MIA) (Travis et al., 2015), eventually developing into invasive adenocarcinoma (IAC). This stratification is critical for patient prognosis and therapeutic decision-making; MIA is defined as having a maximum dimension of the invasive component of ≤5 mm and generally carries an excellent prognosis, whereas IAC exhibits invasion exceeding 5 mm and possesses significant metastatic potential (Zhang et al., 2020). This transition from an indolent to an aggressive phenotype involves complex cellular and molecular reprogramming, including the disruption of the basement membrane and the extensive remodeling of the extracellular matrix (ECM) (Altorki et al., 2019). Deepening our understanding of the molecular drivers facilitating this critical pathological transition is of decisive significance for improving early intervention strategies.

In recent years, advancements in sequencing technologies have revealed that the human lung maintains a dynamic, low-biomass, and highly diverse microbial community shaped by the balance of inhalation, micro-aspiration, and clearance (Tsay et al., 2021). Under pathological conditions, such as chronic respiratory diseases or malignancy, this equilibrium is disrupted, leading to localized dysbiosis. The microbiome within the tumor microenvironment (TME)–comprising bacteria, fungi, and viruses–has emerged as a pivotal dimension of cancer biology (Galeano Niño et al., 2022; Liu et al., 2023). Research indicates that fungal dysbiosis in lung cancer is not a mere bystander effect but plays a functional role in promoting tumor progression by modulating host immune responses, altering metabolic pathways, and enhancing metastatic potential.

As a critical component of the TME, the lung mycobiome is garnering increasing attention for its role in oncogenesis. Evidence suggests that fungal presence can induce chronic inflammation; for instance, certain fungal infections promote the activation of Th17 cells and the production of IL-17, thereby reshaping the TME to favor immunosuppression and immune evasion (Jin et al., 2019; Narunsky-Haziza et al., 2022). Furthermore, specific fungal taxa, such as *Alternaria arborescens* and *Aspergillus* spp., have been found enriched in NSCLC tissues and associated with alterations in the TME (Yang et al., 2025). This intricate interaction between the mycobiome and the host underscores the importance of dissecting how specific fungal communities influence the invasive progression of LUAD.

Based on the context of microbial remodeling during lung cancer progression, this study hypothesizes that the invasive transition from MIA to IAC is driven by specific structural dysbiosis within the lung mycobiome. We propose that these fungal alterations trigger host transcriptomic reprogramming, thereby facilitating tumor invasiveness. To explore this, we employed an integrated multi-omics approach, combining the taxonomic resolution of metagenomic sequencing with the functional insights of bulk RNA-seq to characterize the host response. This framework allows us to bridge ecological observations with molecular mechanisms, providing a high-resolution map of the interaction network between the fungal community and LUAD progression.

## 2 Materials and methods

### 2.1 Study design and participant enrollment

This study enrolled 78 patients with lung adenocarcinoma (LUAD) who underwent surgical resection at the China-Japan Friendship Hospital. To ensure precise clinical phenotyping, pathological staging was determined in strict accordance with the 8th edition of the American Joint Committee on Cancer (AJCC) TNM staging system (Goldstraw et al., 2016). The study protocol underwent rigorous review and was approved by the Ethics Committee of the China-Japan Friendship Hospital (Approval No. ZRJY2021-TD04). Written informed consent was obtained from all participants prior to inclusion.

Patients were strictly stratified into two groups based on pathological invasion depth: the Minimally Invasive Adenocarcinoma group (MIA), defined as tumor size ≤ 3 cm with a maximum stromal invasion diameter of ≤5 mm; and the Invasive Adenocarcinoma group (IAC), defined as having a maximum stromal invasion diameter > 5 mm.

To minimize confounding factors influencing the microbiome, strict inclusion and exclusion criteria were established. Inclusion criteria were: (1) age between 18 and 80 years; (2) histologically confirmed LUAD following surgical resection; and (3) treatment-naive status with no prior anti-tumor therapy. Exclusion criteria included: (1) history of other synchronous or metachronous malignancies; (2) prior neoadjuvant chemotherapy, radiotherapy, or surgical intervention for cancer; (3) use of antibiotics, probiotics, corticosteroids, or immunosuppressants within 1 month preceding enrollment; (4) preoperative CT findings of cavitation or obstructive pneumonia, to exclude secondary fungal colonization; and (5) active pulmonary tuberculosis or systemic fungal infection.

### 2.2 Sample collection and contamination control

Tumor tissues and paired adjacent normal tissues were harvested under aseptic conditions immediately following surgical resection. To prevent environmental and blood-borne microbial contamination, tissues were thoroughly rinsed with sterile saline to remove surface blood and debris. Samples were immediately flash-frozen in liquid nitrogen and stored at −80°C.

Given that lung tissues are low-biomass samples and Metagenomic Next-Generation Sequencing (mNGS) is highly susceptible to environmental contamination, stringent quality control measures were implemented. Blank controls were processed alongside biological samples throughout the nucleic acid extraction and library construction steps to identify and bioinformatically deplete background contaminants.

### 2.3 Multi-omics library construction and sequencing

#### 2.3.1 mNGS and microbiome analysis

To comprehensively characterize the intratumoral fungal community, total DNA was extracted using the QIAamp DNA Microbiome Kit (Qiagen, Germany). To enhance microbial detection sensitivity, the HostZERO Microbial DNA Kit (Zymo Research) was employed during extraction to deplete host DNA. Libraries were constructed using the NEBNext Ultra II DNA Library Prep Kit with DNA fragmented to approximately 350 bp. Whole-genome shotgun sequencing was performed on the Illumina NovaSeq 6000 platform (PE150 mode), targeting a data output of >20 million reads per sample.

#### 2.3.2 Whole exome sequencing (WES) and host variant analysis

To investigate the influence of host genetic background on fungal colonization, genomic DNA was fragmented and subjected to hybrid capture using the Agilent SureSelect Human All Exon V6 Kit. Captured libraries underwent high-depth sequencing on the Illumina NovaSeq 6000 platform, with an average depth of 200× for tumor tissues and 100× for control tissues.

#### 2.3.3 Bulk RNA-seq and host expression profiling

Total RNA was extracted using Trizol Reagent (Invitrogen) and integrity was verified using an Agilent 2100 Bioanalyzer (RIN > 7.0). Eukaryotic mRNA was enriched using Oligo (dT) beads, fragmented, and converted to cDNA. Libraries were constructed using the NEBNext Ultra RNA Library Prep Kit for Illumina (NEB #7530). Following purification and PCR amplification, sequencing was performed on the Illumina NovaSeq X Plus platform (Astrocyte Technology, Hangzhou, China).

### 2.4 Bioinformatics analysis

#### 2.4.1 Microbiome profiling

Raw mNGS data were processed using fastp to remove adapters and low-quality bases (Chen et al., 2018). Clean reads were aligned to the human reference genome (GRCh38) using Bowtie2 to thoroughly remove host sequences. The remaining non-human reads were aligned to a comprehensive microbial database encompassing fungi, bacteria, and viruses using Kraken2 (Wood et al., 2019). Species-level relative abundance was re-estimated using the Bracken algorithm (Lu et al., 2017), focusing on fungal taxa with significant differential abundance between groups.

#### 2.4.2 Transcriptomic data processing

Raw RNA-seq reads were filtered using fastp (v0.23.4) to remove reads containing adapters, >10% N content, or >50% low-quality bases (Q ≤ 20). Clean reads were aligned to the reference genome using HISAT2. Transcripts were assembled and FPKM values calculated using StringTie (v2.2.1). Differential expression analysis was performed using DESeq2, with significance thresholds set at Fold Change (FC) ≥ 2 and *P*-value < 0.05 (Love et al., 2014). Functional annotation and pathway enrichment were conducted using the clusterProfiler package for GO and KEGG analyses. Gene Set Enrichment Analysis (GSEA) was utilized to evaluate metabolic pathway activity differences between groups.

#### 2.4.3 Somatic variant calling

Whole exome sequencing reads were aligned using BWA-MEM. Somatic Single Nucleotide Variants (SNVs) and Indels were identified using the Mutect2 module within GATK (v4.4). Somatic Copy Number Variations (CNVs) were analyzed using CNVkit. Patients were stratified based on the mutation status of common driver genes, including EGFR, KRAS, and TP53, for downstream association analyses.

### 2.5 Survival analysis

To evaluate the prognostic value of fungal-associated hub genes, clinical data and gene expression profiles of Lung Adenocarcinoma (LUAD) patients were retrieved from The Cancer Genome Atlas (TCGA) database. Patients were stratified into high- and low-expression groups based on the median expression value of each candidate hub gene. Survival analysis was performed using the Kaplan-Meier method, and survival differences between groups were assessed using the Log-rank test. The hazard ratio (HR) with 95% confidence intervals (CI) was calculated using the Cox proportional hazards model. A *P*-value < 0.05 was considered statistically significant.

### 2.6 Statistical analysis and integration

All statistical analyses were executed using R software (v4.3.0). Continuous variables were compared using the Wilcoxon rank-sum test, while categorical variables were analyzed using Fisher’s exact test. Spearman rank correlation was used to assess associations between key microbiota and host features (immune infiltration, gene expression), defined by | r| > 0.2 and *P* < 0.05. For WES data, fungal abundance differences between mutant and wild-type groups were analyzed. A two-tailed *P*-value < 0.05 was considered statistically significant. For high-dimensional differential abundance analysis, *P*-values were adjusted for multiple testing using the Benjamini-Hochberg (FDR) method. Given the low-biomass nature of the lung mycobiome, an exploratory significance threshold was defined as a raw *P*-value < 0.05 coupled with an adjusted *P*-value (FDR) < 0.25. Species-level taxa with a prevalence of less than 10% across all samples were excluded from differential abundance analysis to avoid statistical bias.

## 3 Results

### 3.1 Intratumoral fungal community structure in IAC and MIA

**FIGURE 1:**
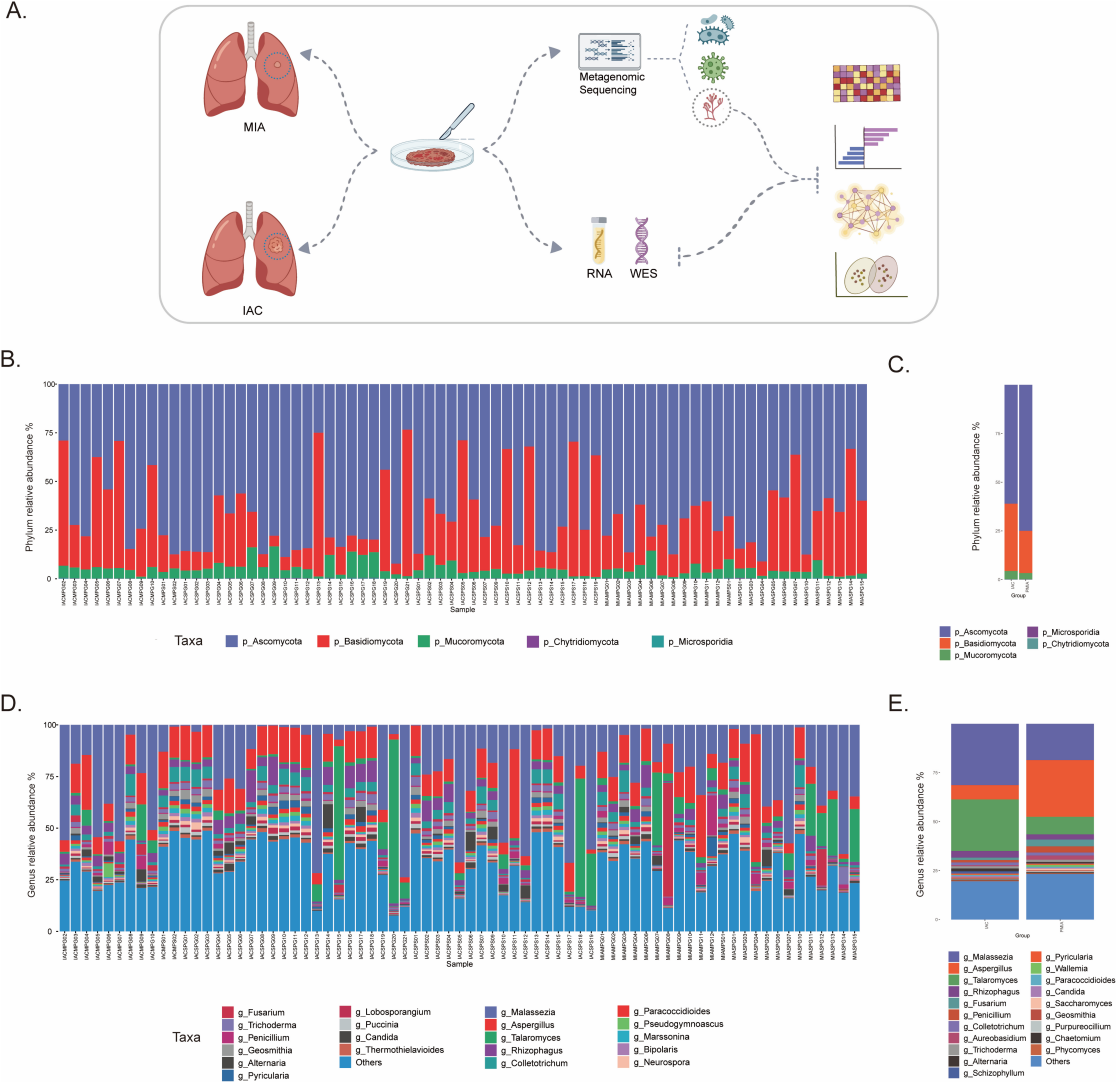
Landscape of the intratumoral fungal community in lung adenocarcinoma subtypes. **(A)** Schematic workflow of the integrated multi-omics study design. Paired tumor (MIA and IAC) and adjacent normal tissues were collected. Total DNA and RNA were extracted from these tissues using specialized microbiome and transcriptomic kits. The analytical pipeline includes metagenomic Next-Generation Sequencing (mNGS) for mycobiome profiling, whole exome sequencing (WES) for host somatic mutation analysis, and Bulk RNA sequencing for host transcriptomic signatures. **(B,C)** Taxonomic composition of the lung mycobiome at the phylum level. **(B)** Phylum relative abundance in individual samples. **(C)** Phylum relative abundance in IAC and MIA groups. **(D,E)** Taxonomic composition at the genus level. **(D)** Genus relative abundance in individual samples. **(E)** Genus relative abundance of dominant fungal genera in IAC and MIA groups.

To investigate the mycobiome dynamics during LUAD progression, we performed multi-omics profiling on paired tumor and adjacent normal tissues (Figure 1A). Metagenomic sequencing and taxonomic annotation of MIA and IAC tissue samples revealed the top 20 fungal taxa at both the individual and group levels. Although fungal distribution varied between samples, a significant difference in community composition was observed between the two groups. At the phylum level, both groups were dominated by Ascomycota and Basidiomycota. In the MIA group, the proportion of Ascomycota was higher, whereas the IAC group showed an enrichment of Basidiomycota (Figures 1B, C, Supplementary Figure 1A). Genus-level analysis identified *Malassezia*, *Aspergillus*, *Talaromyces*, *Rhizophagus*, and *Fusarium* as the five most common intratumoral genera (Figures 1D, E, Supplementary Figure 1B). Notably, *Malassezia* and *Talaromyces* were significantly enriched in the IAC group, while *Aspergillus* was markedly higher in the MIA group, suggesting distinct roles for these genera during tumor progression.

### 3.2 Diversification and key fungal biomarkers identification

**FIGURE 2:**
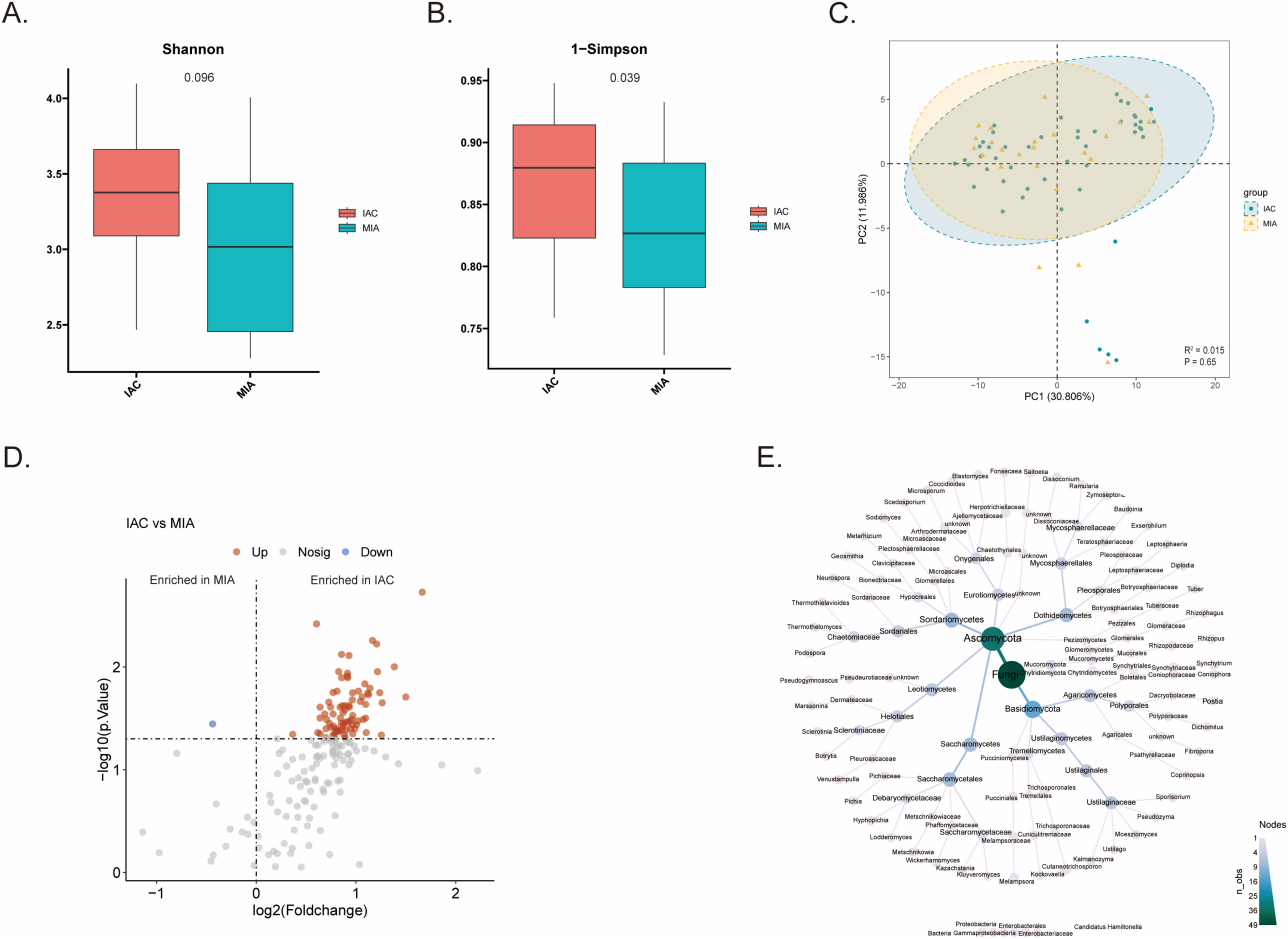
Fungal diversity alterations and identification of *Candida albicans* as an invasive biomarker. **(A,B)** Boxplots comparing fungal alpha-diversity between IAC and MIA groups using the **(A)** Shannon index and **(B)** Simpson index. Significance was tested using the Wilcoxon rank-sum test (*P* < 0.05). **(C)** Principal Coordinate Analysis (PCoA) based on Bray-Curtis dissimilarity showing the β-diversity clustering of fungal communities in MIA and IAC. Statistical significance was assessed using PERMANOVA (R^2^ = 0.015, *P* = 0.65). **(D)** Volcano plot showing differentially abundant fungal genera. Red dots represent genera enriched in IAC, while blue dots represent those enriched in MIA. Statistical significance was determined using the Wilcoxon rank-sum test. **(E)** Taxonomic heat tree illustrating the phylogenetic hierarchy of the fungal community.

Quantitative fungal α-diversity analysis (Shannon and Simpson indices) demonstrated that fungal diversity in the IAC group was significantly higher than in the MIA group (Figures 2A, B), suggesting that the TME fungal ecosystem undergoes a pathological expansion or complexification as LUAD becomes more invasive. However, β-diversity analysis did not show a clear cluster separation between the two groups (PERMANOVA, R^2^ = 0.015, *P* = 0.65), indicating that a complete structural overhaul of the mycobiome had not occurred (Figure 2C). Differential abundance analysis using LDA scores identified *Rhizophagus* as the genus with the highest score (Supplementary Figure 1C). Wilcoxon rank-sum tests identified 82 genera significantly enriched in IAC, while only one genus was enriched in MIA (Figure 2D and Supplementary Table 1). Based on literature review and LEfSe analysis, the genus *Candida* was identified as a critical factor in tumor progression, showing significantly higher abundance in IAC compared to MIA (Supplementary Figure 1D). While *Candida* exhibits strong potential as a biomarker, its relevance was further explored through host transcriptomic and survival analyses. To map the hierarchical lineage of mycobiome dysbiosis, we utilized a taxonomic heat tree (Figure 2E). The taxonomic heat tree highlighted the dominance of the phyla Basidiomycota and Ascomycota, the latter of which encompasses the genus *Candida*. This topological visualization reveals that the significant enrichment of *Candida* in IAC is not an isolated genus-level event, but is deeply rooted within a coordinated expansion of its parent clades within the phylum Ascomycota. Species-level annotation confirmed *s_Candida albicans* as the important contributor to this genus-level enrichment, which was further validated through targeted functional correlations (Supplementary Figures 1E, F).

### 3.3 Transcriptomic divergence between IAC and MIA

**FIGURE 3:**
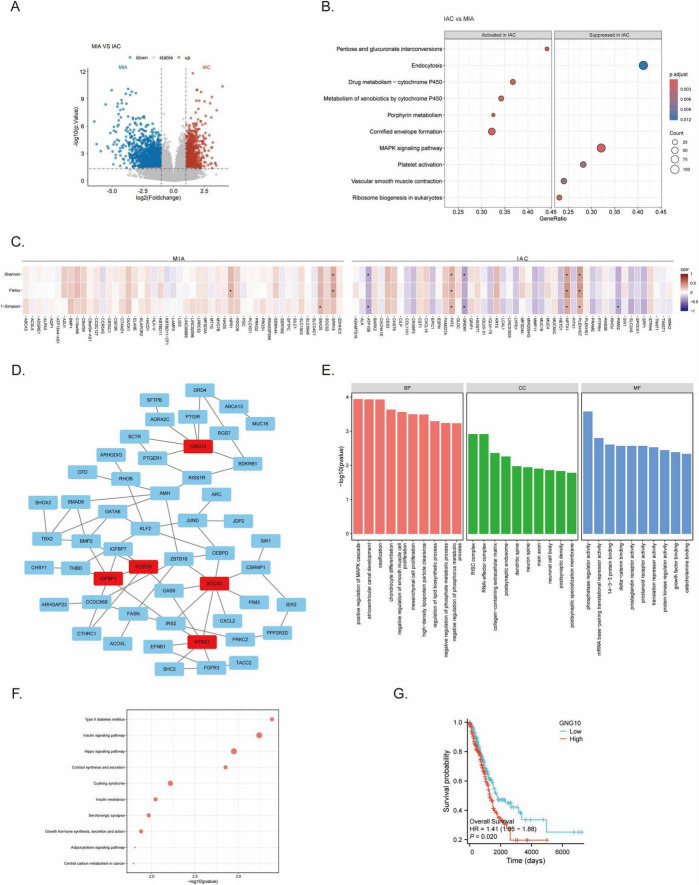
Transcriptomic reprogramming and *Candida albicans*-driven host molecular signatures. **(A)** Volcano plot of Differentially Expressed Genes (DEGs) between IAC and MIA. Red dots represent genes upregulated in IAC, while blue dots represent genes downregulated in IAC. **(B)** GSEA pathway enrichment analysis showing metabolic pathways specifically activated or suppressed in the IAC group. **(C)** Heatmap showing correlations between fungal diversity indices (Shannon/Simpson) and IAC-specific gene signatures. These gene signatures were non-arbitrarily selected by correlating all 2,471 DEGs with diversity indices and filtering for the top highly significant correlations (*P* < 0.05). **(D)** Protein-Protein Interaction (PPI) network of genes significantly associated with *s_Candida albicans*. Red nodes indicate the identified hub genes. **(E,F)** Functional enrichment analysis of *s_Candida albicans*-associated genes. **(E)** Gene Ontology (GO) enrichment. **(F)** KEGG pathway enrichment. **(G)** Kaplan-Meier survival analysis of the hub gene GNG10 in the TCGA-LUAD cohort.

Bulk RNA-seq identified 2,471 differentially expressed genes (DEGs) between MIA and IAC, with 1,574 genes upregulated in IAC and 897 in MIA. Volcano plots illustrated extensive molecular reprogramming during the invasive transition (Figure 3A). GSEA pathway enrichment analysis using the KEGG database revealed that these differences were primarily concentrated in metabolic pathways. Specifically, pathways including pentose and glucuronate interconversions, porphyrin metabolism, and cytochrome P450-related metabolism were significantly enriched and activated in the IAC group compared to the MIA group, highlighting the pivotal role of metabolic shifts in tumor invasiveness (Figure 3B and Supplementary Table 2). To evaluate the clinical relevance of the hub genes, we performed survival analysis using the TCGA-LUAD dataset. High expression of the hub gene GNG10 was significantly associated with poor overall survival (HR = 1.41, *P* = 0.020), providing prognostic evidence for the biological impact of the fungal-associated molecular signatures (Figure 3G).

### 3.4 Identification of *s_Candida albicans*-associated genes and PPI network construction

To determine fungal-driven mechanisms, a systematic correlation analysis was conducted between all 2,471 identified DEGs and fungal α-diversity indices. Characteristic DEGs were strictly defined through a data-driven filtering process: DEGs were evaluated using Spearman correlation, and only those exhibiting statistical significance (*P* < 0.05) alongside the highest absolute correlation coefficients (|ρ|) were selected for visualization. Filtered through this non-arbitrary pipeline, genes such as FAT2, NPTX1, and PLEKHG7 (upregulated signatures of IAC) demonstrated significant positive correlations with fungal diversity (Figure 3C). Correlation analysis also identified *s_Coccidioides posadasii*, *s_Candida albicans*, and *s_Trichoderma asperellum* as key taxa associated with host expression shifts. Specifically, 233 DEGs significantly correlated with *s_Candida albicans* abundance. Although significant species-level abundance differences were limited by the inherent sparsity of reads in low biomass lung tissues, the intersection of differential expression and species-level correlation identifies these 233 genes as a functional bridge linking *s_C. albicans* directly to the IAC specific transcriptomic divergence. This linkage, primarily driven by strong positive associations with pro-invasive markers rather than inhibitory effects, suggests a mechanism of active induction of host malignant reprogramming. To explore the functional interactions of these strictly filtered genes, a protein-protein interaction (PPI) network was constructed (Figure 3D).

### 3.5 Functional enrichment and hub gene identification

To delineate the biological functions of these *s_C. albicans*-associated genes, Gene Ontology (GO) and KEGG enrichment analyses were performed. The results indicated profound activation within the IAC group. Specifically, GO and KEGG enrichment analysis of these genes pointed to two major domains: Metabolism Central carbon metabolism in cancer, phosphatase regulator activity, Insulin signaling and Immunity Hippo signaling, MAPK pathway, cytokine-receptor interaction (Figures 3E, F). These findings suggest that *s_C. albicans* may influence a coordinated immune-metabolic reprogramming in the host. Within the data-driven PPI network, five central hub genes–GNG10, IGFBP3, NTRK1, POSTN, and SOCS3–were identified as core nodes in the fungal-mediated host response. Quantitative correlation analysis confirmed that NTRK1, SOCS3, and GNG10 were positively correlated with *s_Candida albicans* abundance, while IGFBP3 and POSTN were negatively correlated (Supplementary Figure 3). Survival analysis using TCGA-LUAD data revealed that high GNG10 expression is significantly associated with poor prognosis in LUAD patients, while other hub genes showed no significant prognostic association in this cohort (Figure 3G).

### 3.6 Correlation with host immune and metabolic states

**FIGURE 4:**
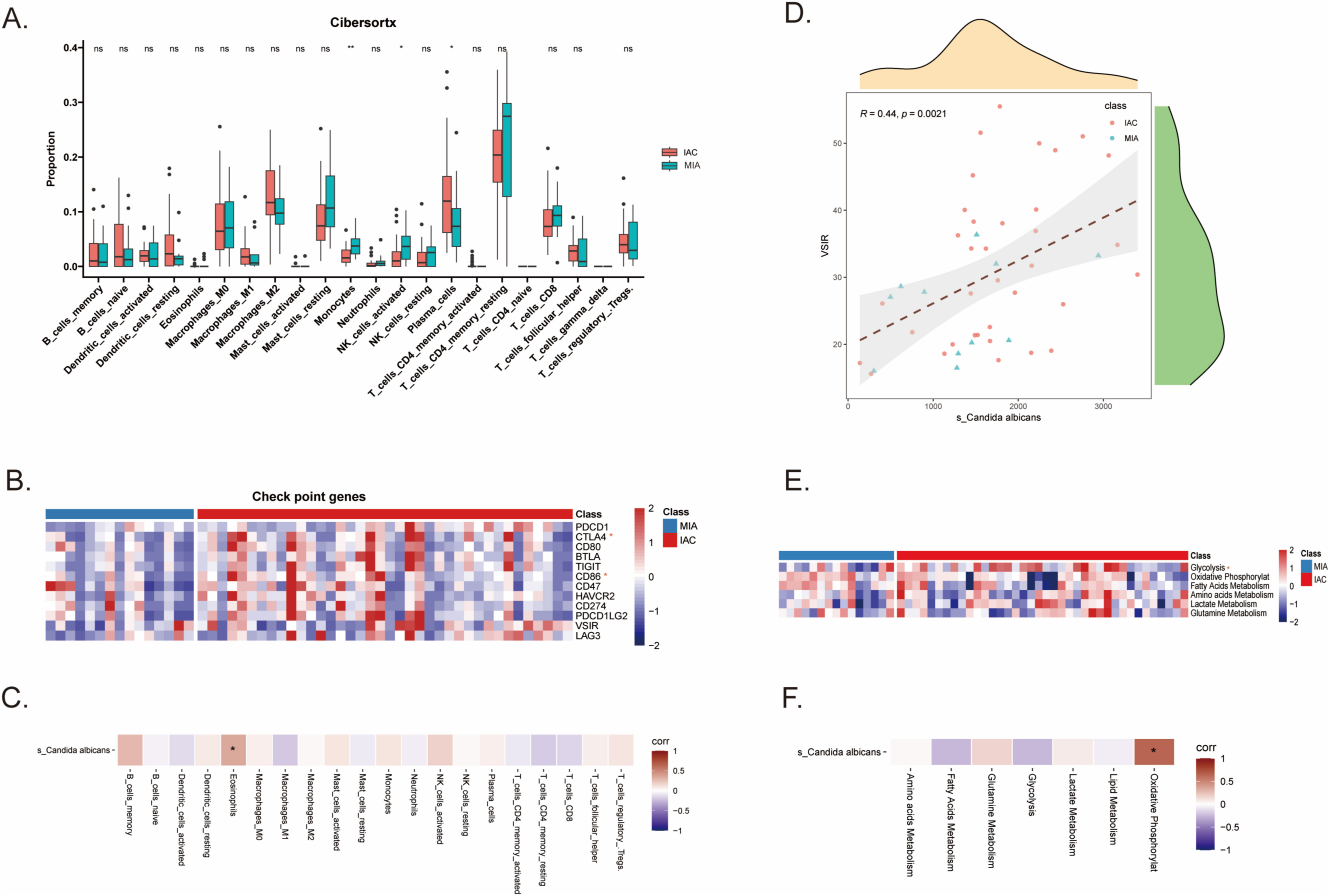
*Candida albicans* orchestrates an immunosuppressive and glycolytic microenvironment. **(A)** Boxplot showing the relative proportions of immune cell types in MIA and IAC estimated by CIBERSORTx. **(B)** Heatmap showing the expression levels of key immune checkpoint genes. **(C)** Correlation analysis showing a significant positive association between *s_Candida albicans* abundance and Eosinophils. **(D)** Scatter plot showing the positive correlation between *s_Candida albicans* abundance and the immune checkpoint VSIR. **(E)** Heatmap comparing metabolic pathway activities between MIA and IAC. **(F)** Correlation heatmap between *s_Candida albicans* and metabolic signatures. Data are presented as mean ± SD. *p < 0.05, **p < 0.01.

CIBERSORT deconvolution revealed that plasma cells were enriched in IAC, while monocytes and activated NK cells were enriched in MIA (Figure 4A). IAC tissues also exhibited higher expression levels of the immune checkpoints CTLA4 and CD86 (Figure 4B). Correlation analysis focused on *s_Candida albicans* revealed a significant positive correlation with eosinophil proportions (Figure 4C) and a close association with the expression of the checkpoint molecule VSIR (Figure 4D). Metabolic analysis confirmed that IAC exhibits significantly higher levels of glycolysis and oxidative phosphorylation than MIA (Figure 4E), and *s_Candida albicans* abundance specifically showed a strong positive correlation with glycolysis (Figure 4F).

### 3.7 Association with clinical features and gene mutations

**FIGURE 5:**
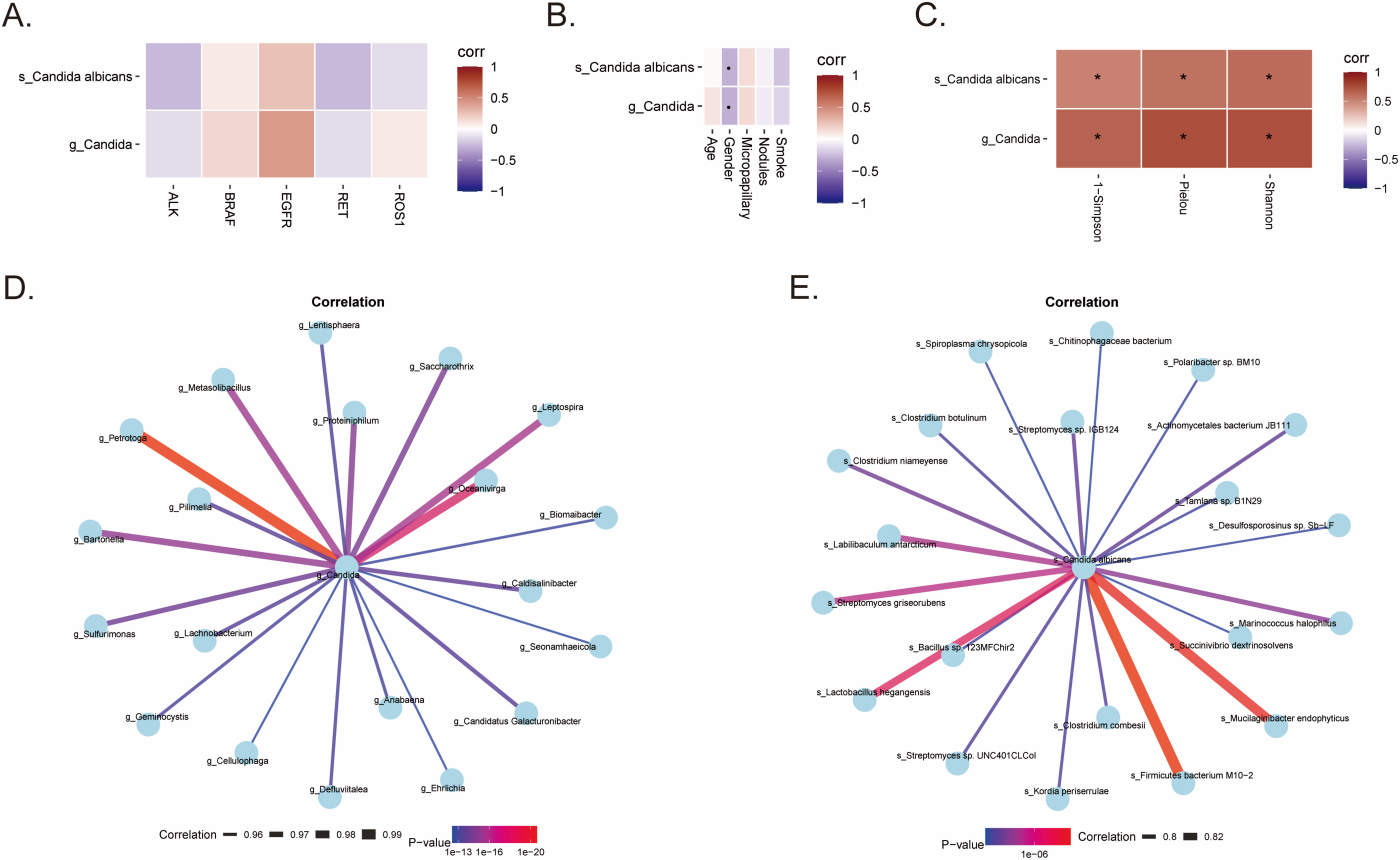
Cross-kingdom interactions and multi-omics correlations. **(A)** Correlation heatmap between *s_Candida albicans/g_Candida* abundance and common driver gene mutations (ALK, BRAF, EGFR, RET, ROS1). **(B)** Correlation heatmap between fungal abundance and clinical features (Age, Gender, Nodules, Smoking). **(C)** Correlation heatmap between fungal abundance (*Candida*) and bacterial α-diversity indices. **(D)** Co-occurrence network of *g_Candida* and bacterial genera in IAC tissues. **(E)** Species-level co-occurrence network of *s_Candida albicans* and specific bacterial species in IAC tissues. Edge width represents correlation strength. Data are presented as mean ± SD. *p < 0.05.

We first investigated the association between the abundance of the genus *Candida* and *s_Candida albicans* and host driver gene mutations. Correlation analysis revealed that both *g_Candida* and *s_Candida albicans* levels were positively associated with several common driver mutations, with the most prominent positive correlation observed with EGFR mutation status (Figure 5A). Analysis of clinical features showed that *g_Candida* and *s_Candida albicans* were partially negatively correlated with patient gender (Corr = −0.29, *P* < 0.05), while no significant associations were found with age, micropapillary structures, nodule count, or smoking history (Figure 5B). At the gene level, *g_Candida* and *s_Candida albicans* were most strongly positively correlated with ANKRD20A5P, CYP4F35P, and ZNF208 (Supplementary Figure 2). Furthermore, genes such as TYW1, CBWD5, and LINC00421 correlated with both the genus and species level (*P* < 0.01), suggesting a common background for fungal-host interactions.

### 3.8 Impact of *g_Candida* and *s_Candida albicans* on bacterial community

We evaluated the relationship between the *Candida* abundance and the bacterial community. Fungal abundance was significantly positively correlated with bacterial α-diversity (Figure 5C), indicating that *Candida* enrichment accompanies a broader bacterial dysbiosis in IAC. Correlation analysis identified *g_Petrotoga* as the bacterial genus most strongly associated with *Candida* in both IAC (Corr = 0.934, Figure 5D) and MIA (Corr = 0.992). Genera like *Oceanivirga* and *Leptospira* also showed extreme correlations (Corr > 0.97) in MIA. Species-level analysis identified *Anaerosalibacter massiliensis* as the strongest associate of *C. albicans* in IAC (Figure 5E), while Firmicutes bacterium M10-2 was the primary associate in MIA (Supplementary Tables 3, 4). *Kordia periserrulae* was identified as a common bacterial partner across both pathological types (Supplementary Tables 3, 4).

## 4 Discussion

This study, through the integration of metagenomic sequencing and transcriptomic analysis, systematically reveals the evolutionary patterns of the fungal community during the transition of LUAD from MIA to IAC and its role in host microenvironmental reconstruction. We observed that the fungal α-diversity of IAC tissue is significantly higher than that of MIA, a finding that challenges the traditional view that disease progression typically leads to a decline in microbial diversity. This pathological expansion of diversity likely reflects the destruction of the tissue basement membrane and the remodeling of the ECM as tumor invasion exceeds 5 mm, creating more complex ecological niches for the colonization of exogenous and symbiotic fungi (Jamal-Hanjani et al., 2017; Nejman et al., 2020; Dejima et al., 2021). At the taxonomic level, the high enrichment of the genus *Candida* in IAC was identified as a key biomarker distinguishing early from invasive LUAD. LEfSe analysis confirmed its high LDA score, suggesting that fungal dysbiosis is not a passive consequence of cancer development but a potential driver of the transition from an indolent to an aggressive phenotype (Tsay et al., 2018; Saftien et al., 2023; Yu et al., 2025).

It is worth noting that in our differential abundance analysis, although certain genera such as *Leptosphaeria* exhibited higher fold changes or more extreme *P*-values than *Candida*, their clinical prevalence in the lung adenocarcinoma microenvironment is poorly documented, and they lacked consistent correlation with host transcriptomic shifts. We prioritized the genus *Candida*, specifically its primary contributor, *s_Candida albicans*, as our primary focus based on its high prevalence in invasive tissues and its well-established pathogenic mechanisms for stromal penetration and immune modulation. Furthermore, the robustness of *Candida* as a key player is supported by its strong and multi-faceted associations with host glycolytic flux and inhibitory immune checkpoint expression, providing a functional rationale that outweighs purely statistical rankings.

Interestingly, while the invasive transition to IAC is marked by a massive enrichment of opportunistic fungi like *Candida*, we observed that only a single genus, *g_Komagataella*, was significantly enriched in the indolent MIA stage. The depletion of this specific early-associated taxon in invasive tissues further underscores the concept of dynamic mycobiome dysbiosis: the disruption and loss of early localized microflora may create a permissive ecological niche, thereby facilitating the subsequent colonization and expansion of pro-tumorigenic fungal communities.

Having colonized this permissive niche, the specific biological characteristics of *s_Candida albicans* strongly support its central role in facilitating tumor invasion. *C. albicans* is an opportunistic pathogen characterized by extreme morphological plasticity, capable of switching between yeast, pseudohyphal, and invasive hyphal forms in response to environmental stress. This transition is accompanied by the secretion of virulence factors such as Candidalysin, which directly causes epithelial damage and physical barrier penetration (Thapa et al., 2025). In the context of IAC pathology, where tumor cells breach the basement membrane to invade the stroma, the mechanism of *C. albicans* active penetration and induced endocytosis shows high spatial consistency with tumor cell behavior. Our correlation analysis further identified a close link between *C. albicans* and genes associated with tumor progression, supporting the hypothesis that this fungus induces a molecular remodeling of host cells, facilitating the shift from an adherent to an invasive growth pattern.

Host transcriptomic features provide further evidence for the mechanism by which *C. albicans* drives tumor progression. We found a significant positive correlation between *C. albicans* abundance and host glycolytic flux, aligning with the classic Warburg effect (Vander Heiden et al., 2009). Even in the presence of oxygen, cancer cells preferentially utilize glycolysis to produce lactate, generating energy and metabolic intermediates required for rapid biomass synthesis (Hanahan, 2022). Literature suggests that *C. albicans* possesses remarkable metabolic flexibility and can compete for glucose in the TME, potentially inducing metabolic exhaustion in host immune cells like macrophages and thereby weakening the antifungal immune response. This fungal-mediated metabolic reprogramming not only provides a proliferative advantage to tumor cells but also creates an acidic environment that further suppresses immune function, establishing a metabolic-immunosuppressive axis that favors tumor diffusion.

Regarding immune microenvironment remodeling, *C. albicans* appears to intervene deeply in host immune checkpoints and immune cell proportions. Our study found that *C. albicans* abundance is closely related to the expression of the checkpoint molecule VSIR. VSIR is a novel inhibitory receptor of the B7 family that, in the acidic TME, can potently induce immune tolerance by limiting antigen presentation and maintaining T cells in a quiescent state (Villarroel-Espindola et al., 2018; Johnston et al., 2019). This positive correlation suggests that *C. albicans* may assist tumor evasion from CD8+ T cell surveillance by inducing VISTA expression within IAC tissues (Chen and Mellman, 2013; Gajewski et al., 2013). Furthermore, the positive correlation between eosinophils and *C. albicans* abundance reveals an additional pathophysiological mechanism. While eosinophils are typically associated with protective antifungal immunity–releasing major basic protein 1 (MBP-1) to inhibit fungal growth–their role in chronic inflammation can be paradoxical. A sustained Th2-mediated inflammatory response can lead to airway remodeling and fibrosis, which may indirectly provide physical conduits for the interstitial invasion of tumor cells (Grisaru-Tal et al., 2021).

The five hub genes identified through PPI network analysis–GNG10, IGFBP3, NTRK1, POSTN, and SOCS3–serve as critical nodes in the fungal-host interaction. The upregulation of GNG10 in IAC and its correlation with poor prognosis suggest it may mediate oncogenic signaling cascades triggered by fungal metabolites (Icgc/Tcga Pan-Cancer Analysis of Whole Genomes Consortium, 2020). SOCS3, a negative regulator of the JAK-STAT pathway, showed a positive correlation with *C. albicans*, potentially reflecting a host compensatory feedback mechanism against excessive fungal-induced chronic inflammation (Jeong et al., 2024). Interestingly, we observed that IGFBP3 and POSTN were negatively correlated with *C. albicans* in our specific cohort. Similarly, the inverse correlation between *C. albicans* and the classic pro-invasive ECM protein *POSTN* (Periostin) presents an intriguing paradox. Given that *POSTN* is predominantly secreted by cancer-associated fibroblasts (CAFs), this negative relationship might indicate spatial heterogeneity within the TME in which fungal colonization and CAF accumulation occupy distinct micro-niches. Alternatively, this could imply that *C. albicans* facilitates stromal invasion through an independent pathway that does not rely on *POSTN*. While IGFBP3 is often described as a tumor suppressor in some lung cancer subsets that sequesters IGF-1 to limit cell survival, its downregulation in *C. albicans*-enriched areas may release this inhibition, synergistically enhancing IGF-mediated LUAD progression. This tissue-specific regulatory pattern highlights the complexity of the TME and warrants further functional validation (Ratajczak-Wielgomas et al., 2016).

Finally, the cross-kingdom interactions revealed between fungi and bacteria offer a new perspective on the lung micro-ecosystem. The positive correlation between *C. albicans* and bacterial diversity, alongside its synergistic enrichment with anaerobic genera such as *Petrotoga* and *Anaerosalibacter massiliensis*, suggests a mutualistic metabolic logic within the microenvironment. Specifically, genuine tumor-associated anaerobes like *A. massiliensis* may produce metabolic byproducts such as lactate that *C. albicans* can utilize as a carbon source, while fungal biofilms, in turn, provide a physical shield for these bacteria against host immune clearance (Shiao et al., 2021). It is worth noting that the extreme environmental taxon *g_Petrotoga* was also detected with high correlation; however, it likely represents a kit or reagent contamination, especially the kitome common in low-biomass sequencing (Salter et al., 2014), and was therefore excluded from further biological interpretation. In conclusion, this study establishes *s_Candida albicans* as a biomarker for LUAD invasive progression and elucidates its multi-faceted potential role associated with tumor evolution through metabolic reconstruction, immune evasion, and cross-kingdom synergy. While bulk RNA-seq has limitations in resolving specific cellular contributors, these findings provide a scientific basis for developing early interventions and combined immunotherapies targeting the lung micro-ecology (Routy et al., 2018).

## 5 Conclusion

By integrating metagenomic and transcriptomic data, this study provides the first comprehensive map of fungal community evolution during the transition of LUAD from MIA to IAC. The results confirm that *s_Candida albicans* is a key driver of this invasive transition, likely promoting a pro-tumorigenic immune-metabolic environment through the upregulation of the VISTA checkpoint, activation of host glycolysis, and synergy with specific anaerobic bacteria. These pathways offer novel biomarkers for distinguishing early from invasive lung adenocarcinoma and represent potential targets for future antifungal-augmented cancer immunotherapies.

## Data Availability

The datasets presented in this study can be found in online repositories. The names of the repository/repositories and accession number(s) can be found in the article/Supplementary material. The original clinical and sequencing contributions (such as mNGS, WES, and Bulk RNA-seq) presented in the study are not readily available due to privacy and ethical restrictions. Requests to access the datasets should be directed to the corresponding author.

## Supplementary material

Supplementary Figure 1 Detailed taxonomic breakdown and differential abundance analysis. **(A,B)** Heatmaps showing the relative abundance of fungal taxa, with values represented as z-scores. **(A)** Clustering heatmap of fungal phyla distribution. **(B)** Clustering heatmap of dominant fungal genera distribution in IAC and MIA groups. **(C)** LEfSe analysis identifying *g_Rhizophagus* as the most differentially abundant feature in IAC. **(D)** Boxplot of *g_Candida* abundance. **(E,F)** Boxplots of species-level abundance for panel **(E)** *s_Candida tropicalis* and **(F)** *s_Candida dubliniensis*, comparing IAC and MIA groups.

Supplementary Figure 2 Additional molecular associations. **(A,B)** Comprehensive correlation analysis between the lung mycobiome and host transcriptomic signatures. **(A)** Genus-level correlation heatmap. **(B)** Species-level correlation heatmap between fungi and host DEGs.

Supplementary Figure 3 Correlation analysis of Hub genes and clinical features. **(A–E)** Scatter plots showing the correlations between *s_Candida albicans* abundance and the expression of five hub genes: **(A)** GNG10, **(B)** IGFBP3, **(C)** NTRK1, **(D)** POSTN, and **(E)** SOCS3. **(F–I)** Associations between fungal abundance and clinical features or host gene mutations.
