# Supplementary material for: Multi-omics landscape of lung mycobiome dysbiosis: Candida albicans drives the invasive progression of lung adenocarcinoma
Source: Front Microbiol. 2026 Apr 15;17:1811749. doi: 10.3389/fmicb.2026.1811749 (PMC13125065; doi:10.3389/fmicb.2026.1811749)
Supplement: Supplementary file 1 [file Table_1.docx]

Supplementary Table S1.：Differential abundance of fungal genera between Invasive Adenocarcinoma (IAC) and Minimally Invasive Adenocarcinoma (MIA) tissues.

| **Name** | **Mean Abundance (IAC)** | **SD (IAC)** | **Mean Abundance (MIA)** | **SD (MIA)** | **p_value** | **p_adjust** | **group** |
| --- | --- | --- | --- | --- | --- | --- | --- |
| *g_Coniophora* | 33.32 | 52.56 | 10.51 | 16.61 | 0.00187 | 0.0972 | IAC |
| *g_Postia* | 11.95 | 9.53 | 7.86 | 12.97 | 0.0038 | 0.115 | IAC |
| *g_Coprinopsis* | 38.02 | 42.68 | 16.95 | 29.3 | 0.00553 | 0.127 | IAC |
| *g_Kluyveromyces* | 53.05 | 62.32 | 22.97 | 41.18 | 0.00597 | 0.129 | IAC |
| *g_Dichomitus* | 11.36 | 11.39 | 6.28 | 8.89 | 0.00755 | 0.137 | IAC |
| *g_Rhizophagus* | 1577.35 | 1850.14 | 827.86 | 1595.53 | 0.00774 | 0.137 | IAC |
| *g_Sporisorium* | 33.71 | 89.39 | 12.91 | 19.65 | 0.00996 | 0.149 | IAC |
| *g_Venustampulla* | 36.32 | 35.3 | 20.48 | 27.95 | 0.0111 | 0.153 | IAC |
| *g_Wickerhamomyces* | 45.13 | 90.59 | 19.43 | 52.14 | 0.0111 | 0.153 | IAC |
| *g_Metschnikowia* | 19.72 | 25.02 | 10.46 | 18.05 | 0.0115 | 0.153 | IAC |
| *g_Botrytis* | 303.66 | 388.67 | 161.18 | 275.86 | 0.0123 | 0.156 | IAC |
| *g_Pseudozyma* | 15.01 | 20.52 | 8.29 | 15.17 | 0.0127 | 0.156 | IAC |
| *g_Podospora* | 178.26 | 234.04 | 94.41 | 173.74 | 0.0127 | 0.156 | IAC |
| *g_Moesziomyces* | 38.6 | 70.13 | 18.15 | 22.79 | 0.0146 | 0.166 | IAC |
| *g_Microsporum* | 41.07 | 51.29 | 18.63 | 29.18 | 0.0161 | 0.173 | IAC |
| *g_Cutaneotrichosporon* | 137.39 | 167.84 | 67.51 | 138.32 | 0.0172 | 0.177 | IAC |
| *g_Melampsora* | 78.29 | 109.14 | 44.65 | 101.39 | 0.0172 | 0.177 | IAC |
| *g_Saitoella* | 39.36 | 54.13 | 23.08 | 52.02 | 0.0177 | 0.179 | IAC |
| *g_Marssonina* | 506.99 | 697.93 | 214.82 | 406.87 | 0.0178 | 0.179 | IAC |
| *g_Candida* | 327.69 | 388.33 | 167.66 | 336.6 | 0.0178 | 0.179 | IAC |
| *g_Synchytrium* | 11.35 | 19.13 | 5.23 | 8.74 | 0.018 | 0.18 | IAC |
| *g_Fibroporia* | 18.6 | 24.86 | 8.52 | 17.84 | 0.0186 | 0.181 | IAC |
| *g_Lodderomyces* | 401.36 | 583.03 | 141.93 | 259.79 | 0.0196 | 0.184 | IAC |
| *g_Hyphopichia* | 46.54 | 67.3 | 22.29 | 55.06 | 0.0196 | 0.184 | IAC |
| *g_Geosmithia* | 811.66 | 1050.66 | 416.05 | 741.65 | 0.0209 | 0.187 | IAC |
| *g_Metarhizium* | 341.2 | 455.39 | 165.53 | 306.17 | 0.0216 | 0.19 | IAC |
| *g_Kazachstania* | 14.49 | 18.44 | 8.75 | 16.45 | 0.022 | 0.192 | IAC |
| *g_Coccidioides* | 306.41 | 453.39 | 127.7 | 203.81 | 0.0223 | 0.192 | IAC |
| *g_Sclerotinia* | 165.81 | 342.27 | 77.51 | 160.37 | 0.0231 | 0.192 | IAC |
| *g_Ustilago* | 17.45 | 25.1 | 9.4 | 12 | 0.0238 | 0.192 | IAC |
| *g_Dissoconium* | 26.7 | 42.25 | 14.67 | 16.12 | 0.0238 | 0.192 | IAC |
| *g_Tuber* | 304.63 | 370.14 | 159.86 | 291.04 | 0.0238 | 0.192 | IAC |
| *g_Exserohilum* | 137.69 | 165.61 | 76.87 | 138.84 | 0.0246 | 0.194 | IAC |
| *g_Leptosphaeria* | 224.64 | 275.25 | 133.94 | 258.46 | 0.0246 | 0.194 | IAC |
| *g_Ramularia* | 19.32 | 22.64 | 10.12 | 15.02 | 0.0246 | 0.194 | IAC |
| *g_Baudoinia* | 12.19 | 11.72 | 7.82 | 10.45 | 0.0246 | 0.194 | IAC |
| *g_Pichia* | 37.59 | 46.15 | 20.28 | 35.72 | 0.0261 | 0.2 | IAC |
| *g_Blastomyces* | 208.94 | 250.19 | 114.24 | 205.78 | 0.0278 | 0.205 | IAC |
| *g_Fonsecaea* | 206.61 | 241.21 | 121.08 | 192.34 | 0.0287 | 0.207 | IAC |
| *g_Zymoseptoria* | 34.34 | 36.31 | 21.34 | 29.15 | 0.0287 | 0.207 | IAC |
| *g_Sodiomyces* | 199.94 | 250.3 | 102.26 | 186.82 | 0.0296 | 0.209 | IAC |
| *g_Kalmanozyma* | 9.69 | 14.25 | 6.22 | 8.99 | 0.0314 | 0.215 | IAC |
| *g_Thermothelomyces* | 289.6 | 406 | 135.45 | 238.66 | 0.0314 | 0.215 | IAC |
| *g_Pseudogymnoascus* | 418.55 | 541.41 | 233.28 | 470.74 | 0.0314 | 0.215 | IAC |
| *g_Thermothielavioides* | 391.39 | 510.29 | 240.11 | 429.01 | 0.0324 | 0.218 | IAC |
| *g_Diplodia* | 148.08 | 171.1 | 76.27 | 130.12 | 0.0324 | 0.218 | IAC |
| *g_Rhizopus* | 62.34 | 77.48 | 29.64 | 57.79 | 0.0324 | 0.218 | IAC |
| *g_Scedosporium* | 174.9 | 211.27 | 86.37 | 142 | 0.0334 | 0.219 | IAC |
| *g_Neurospora* | 435.68 | 562.84 | 227.61 | 399.05 | 0.0334 | 0.219 | IAC |
| *g_Kockovaella* | 39.24 | 53.64 | 20.75 | 37.09 | 0.0344 | 0.22 | IAC |
| *g_Babjeviella* | 13.06 | 15.88 | 7.03 | 11.06 | 0.0344 | 0.22 | IAC |
| *g_Stereum* | 62.74 | 78.88 | 37.7 | 69.42 | 0.0354 | 0.222 | IAC |
| *g_Trichophyton* | 186.56 | 220.63 | 103.34 | 173.38 | 0.0354 | 0.222 | IAC |
| *g_Paracoccidioides* | 597.1 | 722.88 | 325.58 | 610.21 | 0.0354 | 0.222 | IAC |
| *g_Komagataella* | 29.14 | 67.28 | 39.46 | 181.21 | 0.0358 | 0.222 | MIA |
| *g_Verruconis* | 84.23 | 112.92 | 41.95 | 72.69 | 0.0365 | 0.222 | IAC |
| *g_Spizellomyces* | 46.16 | 62.38 | 23.68 | 42.36 | 0.0366 | 0.223 | IAC |
| *g_Anthracocystis* | 79.57 | 89.79 | 46.54 | 79.46 | 0.0376 | 0.224 | IAC |
| *g_Trichosporon* | 20.02 | 41.41 | 10.95 | 15.03 | 0.0376 | 0.224 | IAC |
| *g_Amorphotheca* | 112.46 | 131.52 | 66.98 | 112.44 | 0.0387 | 0.229 | IAC |
| *g_Tilletiaria* | 8.48 | 11.01 | 4.87 | 7.27 | 0.0399 | 0.23 | IAC |
| *g_Trichoderma* | 861.23 | 1040.4 | 447.52 | 702.1 | 0.0399 | 0.23 | IAC |
| *g_Lachnellula* | 72.57 | 93.08 | 44.67 | 73.61 | 0.0399 | 0.23 | IAC |
| *g_Lachancea* | 8.54 | 9.2 | 4.3 | 5.83 | 0.0399 | 0.23 | IAC |
| *g_Puccinia* | 388.62 | 519.63 | 214.11 | 358.52 | 0.041 | 0.233 | IAC |
| *g_Glarea* | 51.93 | 66.43 | 29.45 | 53.73 | 0.041 | 0.233 | IAC |
| *g_Sphaerulina* | 97.04 | 117.46 | 51.99 | 89.59 | 0.041 | 0.233 | IAC |
| *g_Cryptococcus* | 199.13 | 250.78 | 114.77 | 229.31 | 0.0422 | 0.237 | IAC |
| *g_Chaetomium* | 338.74 | 439.16 | 191.79 | 339.35 | 0.0422 | 0.237 | IAC |
| *g_Phycomyces* | 353.96 | 458.22 | 198.5 | 398.28 | 0.0422 | 0.237 | IAC |
| *g_Gloeophyllum* | 23.63 | 31.2 | 15.41 | 28.49 | 0.0435 | 0.237 | IAC |
| *g_Pyricularia* | 657 | 846.28 | 305.93 | 481.28 | 0.0435 | 0.237 | IAC |
| *g_Colletotrichum* | 1725.52 | 2107.3 | 1106.1 | 2241.54 | 0.0447 | 0.238 | IAC |
| *g_Phialemoniopsis* | 80.95 | 99.47 | 41.39 | 61.8 | 0.0447 | 0.238 | IAC |
| *g_Ogataea* | 5.94 | 8.69 | 4.61 | 10.34 | 0.0452 | 0.24 | IAC |
| *g_Acaromyces* | 33.93 | 46.04 | 14.22 | 20.58 | 0.046 | 0.241 | IAC |
| *g_Agaricus* | 37.34 | 40 | 21.89 | 37.05 | 0.046 | 0.241 | IAC |
| *g_Kwoniella* | 155.53 | 172.44 | 84.21 | 135.68 | 0.046 | 0.241 | IAC |
| *g_Phialocephala* | 66.97 | 82.1 | 38.98 | 70.96 | 0.046 | 0.241 | IAC |
| *g_Cordyceps* | 107.12 | 111.65 | 66.04 | 94.16 | 0.0487 | 0.249 | IAC |
| *g_Lindgomyces* | 98.25 | 110.87 | 57.09 | 97.38 | 0.0487 | 0.249 | IAC |
| *g_Clavispora* | 92.55 | 115.17 | 49.94 | 81.94 | 0.0487 | 0.249 | IAC |
