# Supplementary material for: Multi-omics landscape of lung mycobiome dysbiosis: Candida albicans drives the invasive progression of lung adenocarcinoma
Source: Front Microbiol. 2026 Apr 15;17:1811749. doi: 10.3389/fmicb.2026.1811749 (PMC13125065; doi:10.3389/fmicb.2026.1811749)
Supplement: Supplementary file 2 [file Table_2.docx]

**Supplementary Table 2. GSEA enrichment analysis of metabolic and signaling pathways associated with IAC.**

| **ID** | **Description** | **setSize** | **enrichmentScore** | **NES** | **pvalue** | **p.adjust** | **qvalue** | **rank** | **leading_edge** |
| --- | --- | --- | --- | --- | --- | --- | --- | --- | --- |
| hsa04382 | Cornified envelope formation | 180 | 0.526217378 | 1.868349177 | 3.44E-07 | 0.000120201 | 0.000102237 | 4934 | tags=32%, list=15%, signal=27% |
| hsa03008 | Ribosome biogenesis in eukaryotes | 104 | -0.497476437 | -2.033668504 | 1.06E-06 | 0.000184992 | 0.000157345 | 2562 | tags=23%, list=8%, signal=21% |
| hsa04010 | MAPK signaling pathway | 299 | -0.352062576 | -1.63268433 | 5.51E-06 | 0.000641298 | 0.000545456 | 6458 | tags=32%, list=20%, signal=26% |
| hsa00040 | Pentose and glucuronate interconversions | 34 | 0.718863998 | 2.040455433 | 8.86E-06 | 0.000773039 | 0.000657509 | 3127 | tags=44%, list=10%, signal=40% |
| hsa00980 | Metabolism of xenobiotics by cytochrome P450 | 73 | 0.601055655 | 1.923273296 | 1.38E-05 | 0.000964297 | 0.000820183 | 3844 | tags=34%, list=12%, signal=30% |
| hsa00860 | Porphyrin metabolism | 43 | 0.673961519 | 1.978138897 | 2.51E-05 | 0.00125765 | 0.001069695 | 3127 | tags=33%, list=10%, signal=29% |
| hsa00982 | Drug metabolism - cytochrome P450 | 68 | 0.606963239 | 1.9292454 | 2.52E-05 | 0.00125765 | 0.001069695 | 3844 | tags=37%, list=12%, signal=32% |
| hsa05322 | Systemic lupus erythematosus | 133 | 0.503145813 | 1.743886775 | 0.000100892 | 0.004401431 | 0.003743639 | 5747 | tags=40%, list=18%, signal=33% |
| hsa04611 | Platelet activation | 124 | -0.414328877 | -1.746100495 | 0.00014646 | 0.005679411 | 0.004830625 | 6186 | tags=28%, list=19%, signal=23% |
| hsa04270 | Vascular smooth muscle contraction | 133 | -0.38753915 | -1.647682524 | 0.000205764 | 0.007096372 | 0.006035822 | 4272 | tags=24%, list=13%, signal=21% |
| hsa04740 | Olfactory transduction | 220 | 0.44019768 | 1.591908506 | 0.000223668 | 0.007096372 | 0.006035822 | 5220 | tags=30%, list=16%, signal=25% |
| hsa05204 | Chemical carcinogenesis - DNA adducts | 66 | 0.561302875 | 1.781362984 | 0.000328013 | 0.008520868 | 0.007247428 | 3789 | tags=30%, list=12%, signal=27% |
| hsa00983 | Drug metabolism - other enzymes | 78 | 0.540845039 | 1.745406459 | 0.000341811 | 0.008520868 | 0.007247428 | 3789 | tags=28%, list=12%, signal=25% |
| hsa05034 | Alcoholism | 186 | 0.45059056 | 1.608246559 | 0.000308771 | 0.008520868 | 0.007247428 | 5572 | tags=33%, list=17%, signal=27% |
| hsa00140 | Steroid hormone biosynthesis | 60 | 0.581262486 | 1.818758709 | 0.000382164 | 0.008891677 | 0.00756282 | 3502 | tags=32%, list=11%, signal=28% |
| hsa04144 | Endocytosis | 250 | -0.321222654 | -1.481701933 | 0.000558555 | 0.012183479 | 0.010362663 | 8415 | tags=41%, list=26%, signal=31% |
| hsa00830 | Retinol metabolism | 65 | 0.552509166 | 1.748390308 | 0.000696232 | 0.014293241 | 0.012157122 | 3127 | tags=31%, list=10%, signal=28% |
| hsa00053 | Ascorbate and aldarate metabolism | 28 | 0.673178306 | 1.842505909 | 0.001412365 | 0.026354899 | 0.022416171 | 3127 | tags=46%, list=10%, signal=42% |
| hsa04340 | Hedgehog signaling pathway | 56 | -0.476262499 | -1.736500963 | 0.001434794 | 0.026354899 | 0.022416171 | 9787 | tags=50%, list=30%, signal=35% |
| hsa04370 | VEGF signaling pathway | 59 | -0.469350921 | -1.706105519 | 0.001662677 | 0.029013705 | 0.02467762 | 6458 | tags=34%, list=20%, signal=27% |
| hsa04082 | Neuroactive ligand signaling | 195 | 0.416807381 | 1.495205972 | 0.00256784 | 0.039688021 | 0.033756664 | 4375 | tags=27%, list=13%, signal=23% |
| hsa05224 | Breast cancer | 147 | -0.340652692 | -1.456624315 | 0.002591752 | 0.039688021 | 0.033756664 | 6670 | tags=33%, list=21%, signal=27% |
| hsa04015 | Rap1 signaling pathway | 210 | -0.313340457 | -1.410664427 | 0.002615543 | 0.039688021 | 0.033756664 | 7008 | tags=32%, list=22%, signal=26% |
| hsa00220 | Arginine biosynthesis | 23 | -0.594853838 | -1.762728062 | 0.002794452 | 0.040635983 | 0.034562954 | 4775 | tags=43%, list=15%, signal=37% |
| hsa04722 | Neurotrophin signaling pathway | 119 | -0.361631893 | -1.507433217 | 0.003219124 | 0.044938967 | 0.038222859 | 10247 | tags=45%, list=32%, signal=31% |
| hsa04924 | Renin secretion | 69 | -0.425564653 | -1.608730208 | 0.003451277 | 0.046326753 | 0.03940324 | 3525 | tags=28%, list=11%, signal=25% |
| hsa04910 | Insulin signaling pathway | 136 | -0.341550942 | -1.446500631 | 0.004229129 | 0.052354187 | 0.044529877 | 3975 | tags=30%, list=12%, signal=27% |
| hsa04072 | Phospholipase D signaling pathway | 146 | -0.329919873 | -1.407008379 | 0.004071338 | 0.052354187 | 0.044529877 | 7529 | tags=37%, list=23%, signal=29% |
| hsa04151 | PI3K-Akt signaling pathway | 345 | -0.272198761 | -1.275835642 | 0.004350348 | 0.052354187 | 0.044529877 | 4552 | tags=22%, list=14%, signal=19% |
| hsa04664 | Fc epsilon RI signaling pathway | 66 | -0.414828427 | -1.54705735 | 0.005535124 | 0.062234426 | 0.052933519 | 6458 | tags=29%, list=20%, signal=23% |
| hsa04071 | Sphingolipid signaling pathway | 121 | -0.353664735 | -1.486067419 | 0.005538376 | 0.062234426 | 0.052933519 | 6778 | tags=29%, list=21%, signal=23% |
| hsa05222 | Small cell lung cancer | 92 | -0.367661179 | -1.485094939 | 0.006241275 | 0.062234426 | 0.052933519 | 5712 | tags=27%, list=18%, signal=22% |
| hsa04935 | Growth hormone synthesis, secretion and action | 116 | -0.347399782 | -1.44487157 | 0.006092693 | 0.062234426 | 0.052933519 | 2970 | tags=27%, list=9%, signal=24% |
| hsa04080 | Neuroactive ligand-receptor interaction | 345 | 0.36416895 | 1.369252621 | 0.005928199 | 0.062234426 | 0.052933519 | 4639 | tags=26%, list=14%, signal=23% |
| hsa04014 | Ras signaling pathway | 235 | -0.291951966 | -1.332605864 | 0.005893765 | 0.062234426 | 0.052933519 | 6482 | tags=30%, list=20%, signal=24% |
| hsa04923 | Regulation of lipolysis in adipocytes | 57 | -0.4261395 | -1.552608878 | 0.006642907 | 0.064399293 | 0.054774848 | 6533 | tags=39%, list=20%, signal=31% |
| hsa04931 | Insulin resistance | 107 | -0.353739658 | -1.447259794 | 0.007288133 | 0.066935747 | 0.056932229 | 5802 | tags=24%, list=18%, signal=20% |
| hsa04022 | cGMP-PKG signaling pathway | 165 | -0.311346728 | -1.365923108 | 0.007253074 | 0.066935747 | 0.056932229 | 3534 | tags=19%, list=11%, signal=17% |
| hsa00532 | Glycosaminoglycan biosynthesis - chondroitin sulfate / dermatan sulfate | 21 | -0.58760698 | -1.720796928 | 0.008015088 | 0.071724766 | 0.061005531 | 10684 | tags=76%, list=33%, signal=51% |
| hsa04110 | Cell cycle | 158 | 0.41733842 | 1.46723426 | 0.009175768 | 0.080058577 | 0.068093858 | 8467 | tags=37%, list=26%, signal=28% |
| hsa05217 | Basal cell carcinoma | 63 | -0.398214383 | -1.47676134 | 0.009815734 | 0.083553446 | 0.071066421 | 6666 | tags=41%, list=20%, signal=33% |
| hsa04933 | AGE-RAGE signaling pathway in diabetic complications | 100 | -0.353427989 | -1.436039747 | 0.01105667 | 0.090655059 | 0.077106701 | 6224 | tags=32%, list=19%, signal=26% |
| hsa03040 | Spliceosome | 155 | -0.315423584 | -1.372337291 | 0.011169535 | 0.090655059 | 0.077106701 | 2094 | tags=10%, list=6%, signal=9% |
| hsa01240 | Biosynthesis of cofactors | 152 | 0.409032163 | 1.438066715 | 0.012816036 | 0.101654469 | 0.086462254 | 4386 | tags=17%, list=13%, signal=15% |
| hsa04330 | Notch signaling pathway | 62 | -0.406767481 | -1.50295652 | 0.017904215 | 0.13696432 | 0.116495064 | 5711 | tags=31%, list=18%, signal=25% |
| hsa04115 | p53 signaling pathway | 75 | 0.453203844 | 1.454587199 | 0.018052604 | 0.13696432 | 0.116495064 | 3458 | tags=19%, list=11%, signal=17% |
| hsa04912 | GnRH signaling pathway | 91 | -0.338106759 | -1.354614708 | 0.020478966 | 0.152067218 | 0.12934084 | 6685 | tags=30%, list=21%, signal=24% |
| hsa05150 | Staphylococcus aureus infection | 80 | 0.452623745 | 1.470368914 | 0.021422039 | 0.155756077 | 0.132478401 | 3984 | tags=24%, list=12%, signal=21% |
| hsa05220 | Chronic myeloid leukemia | 76 | -0.368416093 | -1.424100033 | 0.022868948 | 0.159625255 | 0.135769331 | 11048 | tags=47%, list=34%, signal=31% |
| hsa04650 | Natural killer cell mediated cytotoxicity | 116 | -0.323873562 | -1.34702359 | 0.022611125 | 0.159625255 | 0.135769331 | 6685 | tags=25%, list=21%, signal=20% |
| hsa04630 | JAK-STAT signaling pathway | 149 | -0.29973234 | -1.288554725 | 0.023332123 | 0.159664918 | 0.135803067 | 4599 | tags=19%, list=14%, signal=16% |
| hsa01521 | EGFR tyrosine kinase inhibitor resistance | 79 | -0.353118247 | -1.383540263 | 0.023825807 | 0.159907823 | 0.13600967 | 6458 | tags=27%, list=20%, signal=21% |
| hsa04510 | Focal adhesion | 202 | -0.287438428 | -1.288524495 | 0.024635035 | 0.162219382 | 0.137975768 | 6458 | tags=31%, list=20%, signal=25% |
| hsa04625 | C-type lectin receptor signaling pathway | 104 | -0.325536835 | -1.330784652 | 0.030894585 | 0.199670561 | 0.169829884 | 6593 | tags=27%, list=20%, signal=22% |
| hsa01522 | Endocrine resistance | 96 | -0.327458815 | -1.326147653 | 0.032717219 | 0.207605623 | 0.176579055 | 6593 | tags=29%, list=20%, signal=23% |
| hsa03440 | Homologous recombination | 41 | 0.511504543 | 1.487770933 | 0.033578779 | 0.209267746 | 0.177992774 | 10306 | tags=59%, list=32%, signal=40% |
| hsa04610 | Complement and coagulation cascades | 82 | -0.342809629 | -1.351785318 | 0.034649618 | 0.212152924 | 0.180446764 | 4459 | tags=33%, list=14%, signal=28% |
| hsa03030 | DNA replication | 36 | 0.531668927 | 1.520011518 | 0.035880566 | 0.213833591 | 0.181876256 | 10381 | tags=61%, list=32%, signal=42% |
| hsa05231 | Choline metabolism in cancer | 98 | -0.322214331 | -1.306009642 | 0.036149518 | 0.213833591 | 0.181876256 | 6532 | tags=24%, list=20%, signal=20% |
| hsa05216 | Thyroid cancer | 37 | -0.43259931 | -1.438389103 | 0.037162117 | 0.216159648 | 0.183854685 | 4253 | tags=43%, list=13%, signal=38% |
| hsa04742 | Taste transduction | 80 | 0.43125902 | 1.400964628 | 0.037908497 | 0.216886317 | 0.184472754 | 7266 | tags=35%, list=22%, signal=27% |
| hsa04668 | TNF signaling pathway | 118 | -0.309050735 | -1.282222307 | 0.039099813 | 0.220094108 | 0.187201142 | 5713 | tags=25%, list=18%, signal=21% |
| hsa04917 | Prolactin signaling pathway | 68 | -0.350702639 | -1.314871874 | 0.039757984 | 0.220246609 | 0.187330852 | 6675 | tags=29%, list=21%, signal=23% |
| hsa00790 | Folate biosynthesis | 28 | 0.562867398 | 1.540582187 | 0.042002075 | 0.228204585 | 0.194099511 | 2875 | tags=21%, list=9%, signal=20% |
| hsa04012 | ErbB signaling pathway | 85 | -0.318664655 | -1.260015756 | 0.042502287 | 0.228204585 | 0.194099511 | 10399 | tags=45%, list=32%, signal=30% |
| hsa04976 | Bile secretion | 87 | 0.425831977 | 1.401764023 | 0.043367347 | 0.229321274 | 0.195049312 | 4699 | tags=28%, list=14%, signal=24% |
| hsa03010 | Ribosome | 157 | -0.289384591 | -1.256607484 | 0.045120482 | 0.235030568 | 0.199905354 | 2562 | tags=14%, list=8%, signal=13% |
