# Supplementary material for: Multi-omics landscape of lung mycobiome dysbiosis: Candida albicans drives the invasive progression of lung adenocarcinoma
Source: Front Microbiol. 2026 Apr 15;17:1811749. doi: 10.3389/fmicb.2026.1811749 (PMC13125065; doi:10.3389/fmicb.2026.1811749)
Supplement: Supplementary file 3 [file Table_3.docx]

| **node1** | **node2** | **corr** | **pvalue** | **label** |
| --- | --- | --- | --- | --- |
| g_Candida | g_Petrotoga | 0.927046942695222 | 1.17904970396062e-21 | *** |
| g_Candida | g_Bartonella | 0.918684022872566 | 1.37564279363465e-20 | *** |
| g_Candida | g_Butyrivibrio | 0.910995575871938 | 1.05448705887485e-19 | *** |
| g_Candida | g_Photorhabdus | 0.899602012620519 | 1.57244258908632e-18 | *** |
| g_Candida | g_Leptospira | 0.89258961635102 | 7.09756373846003e-18 | *** |
| g_Candida | g_Companilactobacillus | 0.891810474792673 | 8.33743383669838e-18 | *** |
| g_Candida | g_Brachyspira | 0.886282709144221 | 2.52523756469453e-17 | *** |
| g_Candida | g_Francisella | 0.879943540233858 | 8.4050742159611e-17 | *** |
| g_Candida | g_Arenibacter | 0.878865925413354 | 1.02421981465093e-16 | *** |
| g_Candida | g_Flammeovirga | 0.87840509600788 | 1.11393119397402e-16 | *** |
| g_Candida | g_Candidatus Izimaplasma | 0.87773249734582 | 1.25839510816582e-16 | *** |
| g_Candida | g_Paraglaciecola | 0.870610856862374 | 4.38712364396621e-16 | *** |
| g_Candida | g_Anaerosalibacter | 0.868260310155305 | 6.51931292792438e-16 | *** |
| g_Candida | g_Algibacter | 0.864057392243132 | 1.29935585434029e-15 | *** |
| g_Candida | g_Psychrilyobacter | 0.861691238341829 | 1.89672292567374e-15 | *** |
| g_Candida | g_Seonamhaeicola | 0.859249525732905 | 2.78214786666983e-15 | *** |
| g_Candida | g_Oceanivirga | 0.852152363110413 | 8.14369905706764e-15 | *** |
| g_Candida | g_Methylacidiphilum | 0.851751246638657 | 8.63885437384573e-15 | *** |
| g_Candida | g_Ehrlichia | 0.850736760702601 | 1.00220213778546e-14 | *** |
| g_Candida | g_Massilimicrobiota | 0.850627581841098 | 1.0182826723761e-14 | *** |
