# Supplementary material for: Multi-omics landscape of lung mycobiome dysbiosis: Candida albicans drives the invasive progression of lung adenocarcinoma
Source: Front Microbiol. 2026 Apr 15;17:1811749. doi: 10.3389/fmicb.2026.1811749 (PMC13125065; doi:10.3389/fmicb.2026.1811749)
Supplement: Supplementary file 4 [file Table_4.docx]

| **node1** | **node2** | **corr** | **pvalue** | **label** |
| --- | --- | --- | --- | --- |
| s_Candida albicans | s_Streptomyces sp. NRRL S-1448 | 0.908425658339195 | 1.99979990759155e-19 | *** |
| s_Candida albicans | s_Serinicoccus sediminis | 0.893487596024727 | 5.88644086720484e-18 | *** |
| s_Candida albicans | s_Clostridium magnum | 0.892751568785664 | 6.86290110962579e-18 | *** |
| s_Candida albicans | s_Anaerosalibacter massiliensis | 0.873735276441144 | 2.56015404852673e-16 | *** |
| s_Candida albicans | s_Rhodococcus defluvii | 0.871241499808423 | 3.93964133741368e-16 | *** |
| s_Candida albicans | s_Helicobacter valdiviensis | 0.864675327883637 | 1.17576182377931e-15 | *** |
| s_Candida albicans | s_Clostridium amylolyticum | 0.863058473822239 | 1.52564335370631e-15 | *** |
| s_Candida albicans | s_Streptomyces niger | 0.860049587281833 | 2.45589536781097e-15 | *** |
| s_Candida albicans | s_Streptomyces resistomycificus | 0.854269390960456 | 5.94656925063038e-15 | *** |
| s_Candida albicans | s_Streptomyces sp. ST1020 | 0.849618500960252 | 1.178971433918e-14 | *** |
| s_Candida albicans | s_Formosa agariphila | 0.84824625669018 | 1.43648096337236e-14 | *** |
| s_Candida albicans | s_Mesorhizobium sp. M7A.F.Ca.CA.001.13.1.1 | 0.846807632173486 | 1.7633800338348e-14 | *** |
| s_Candida albicans | s_Chryseobacterium sp. KBW03 | 0.84273833379131 | 3.11440376821305e-14 | *** |
| s_Candida albicans | s_Streptomyces sp. XY431 | 0.842271216269079 | 3.32112226112393e-14 | *** |
| s_Candida albicans | s_Myxococcus sp. CA023 | 0.836394868156867 | 7.3260549205414e-14 | *** |
| s_Candida albicans | s_Actinobacteria bacterium OK006 | 0.835963235663885 | 7.7548438353799e-14 | *** |
| s_Candida albicans | s_Algibacter sp. L1A34 | 0.835561253003253 | 8.17551402865784e-14 | *** |
| s_Candida albicans | s_Streptomyces griseorubens | 0.834647098537582 | 9.21424441041694e-14 | *** |
| s_Candida albicans | s_Myxococcus sp. AM001 | 0.833219363691621 | 1.10907373621411e-13 | *** |
| s_Candida albicans | s_Streptomyces sp. NRRL WC-3725 | 0.829772037702507 | 1.72287158952643e-13 | *** |
