# Supplementary figures and images for: Multi-omics landscape of lung mycobiome dysbiosis: Candida albicans drives the invasive progression of lung adenocarcinoma
Source: Front Microbiol. 2026 Apr 15;17:1811749. doi: 10.3389/fmicb.2026.1811749 (PMC13125065; doi:10.3389/fmicb.2026.1811749)

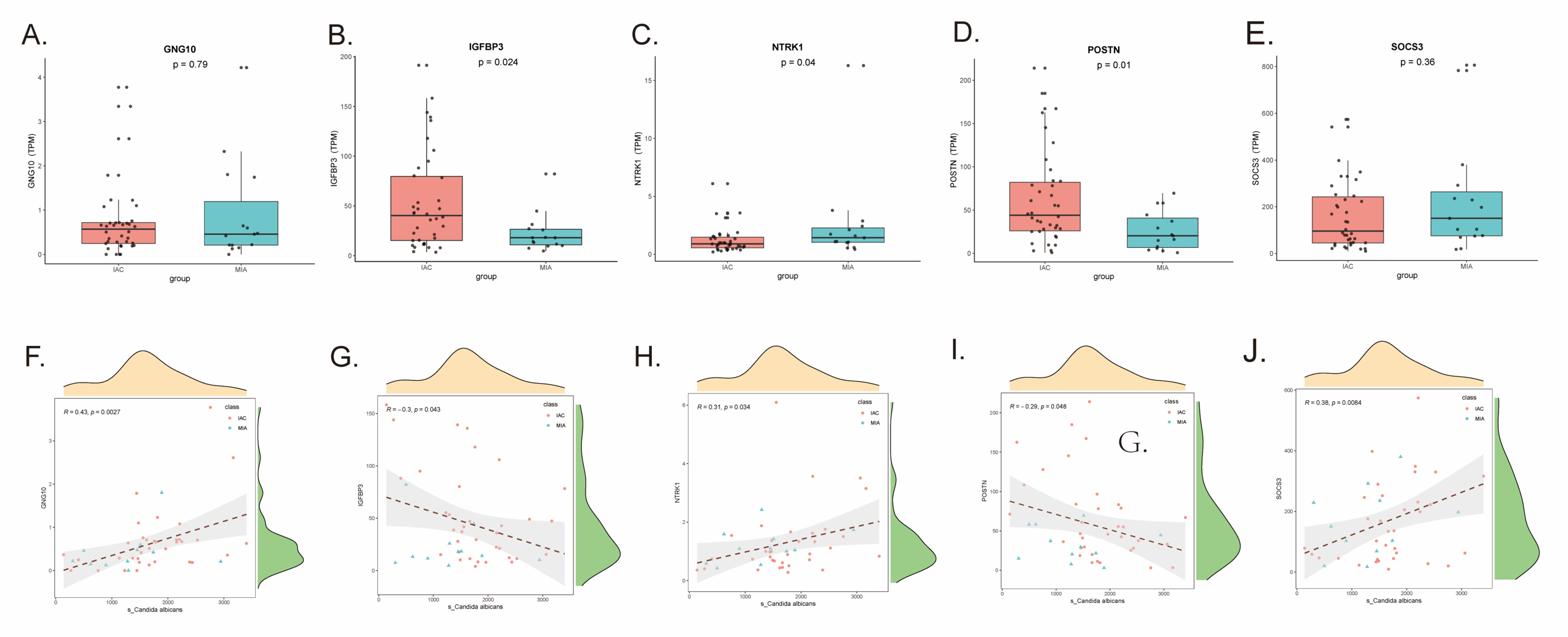

Supplement: Supplementary Figure 3 — Correlation analysis of Hub genes and clinical features. (A–E) Scatter plots showing the correlations between s_Candida albicans abundance and the expression of five hub genes: (A) GNG10, (B) IGFBP3, (C) NTRK1, (D) POSTN, and (E) SOCS3. (F–I) Associations between fungal abundance and clinical features or host gene mutations. [file Image_3.tif]
